# Epididymo-Orchitis and Pelvic Abscess in a Patient With Inflatable Penile Prosthesis

**DOI:** 10.7759/cureus.29715

**Published:** 2022-09-28

**Authors:** Miyaz Ansari, Rohan K Mangal, Thor S Stead, Matthew Carman, Latha Ganti

**Affiliations:** 1 Biology, Steinbrenner High School, Lutz, USA; 2 Medicine, University of Miami Miller School of Medicine, Miami, USA; 3 Medicine, Warren Alpert Medical School of Brown University, Providence, USA; 4 Emergency Medicine, Lakeland Regional Health, Lakeland, USA; 5 Emergency Medicine, HCA Florida Ocala Hospital, Ocala, USA; 6 Emergency Medicine, Envision Physician Services, Plantation, USA; 7 Emergency Medicine, University of Central Florida College of Medicine, Orlando, USA

**Keywords:** prosthetic infection, urethral injury, penile prosthesis, emergency medicine, epididymo-orchitis

## Abstract

This report discusses a case of a 79-year-old male with a urethral injury following implantation of an inflatable penile prosthesis, leading to frequent urinary tract infections. The patient presented with scrotal pain and swelling, as well as abdominal and suprapubic tenderness and rebound. He was diagnosed with epididymo-orchitis, penile prosthetic infection, and pelvis abscess, and was treated with cephalexin. The patient was admitted for urology consultation and had an uneventful hospital stay.

## Introduction

Epididymo-orchitis (EO) is the inflammation of both the epididymis and ipsilateral testis. This condition is uncommon, with 600,000 reported cases of epididymitis per year in the United States, and primarily affects men between 18 and 35 years of age [1]. Orchitis is even more rare; one outpatient study found that orchitis occurs in 58% of cases where men have epididymitis [2]. EO can be caused by pathogens, bacterial or viral, with the most common being *Chlamydia trachomatis* and *Neisseria gonorrhoeae*. Men younger than 14 years and older than 35 years are less likely to have EO due to bacteria that are sexually transmitted and are usually infected by common urinary tract pathogens such as *Escherichia coli* [2]. Men with EO most often present with chief complaints of scrotal pain and symptoms of lower urinary tract infection (UTI).

Risk factors for EO include a history of epididymitis, unprotected sexual intercourse, extended use of a Foley catheter, bladder outlet obstruction, and lacking immunization against measles, mumps, and rubella. Cases of EO have also been attributed to urinary instrumentation [1]. Physical examination can help distinguish cases of EO from testicular torsion, which occurs far more often. While acute pain in the testicles and scrotum are symptoms shared between EO and torsion, the UTI symptoms found in EO cases including fever, hematuria, and dysuria are unlikely with testicular torsion. Furthermore, the inguinal area can be searched for hernia or tender lymph nodes, which would suggest an inflammatory response at play, indicating epididymitis or orchitis. Another physical examination finding may be a tender spermatic cord, confirming epididymitis [3].

When physical examination findings are unclear or additional testing is needed, a gram stain and culture of urethral discharge is recommended. Polymerase chain reaction assays for the sample can then be used to determine the pathogen causing the infection. An ultrasound can also be used to visualize Doppler wave pulsation. Ultrasonography provides information on whether blood flow has been restricted. In this situation, physicians can more easily attribute the patient’s pain to testicular torsion. Conversely, if there is an increase in wave pulsation, this would suggest an enlarged epididymis or epididymitis [4,5]. The urgency to diagnose EO is important; if left untreated, EO can lead to infertility.

Penile prostheses are a viable option for patients with erectile dysfunction. These devices allow for penetrative sexual activity without impacting the ability to urinate or ejaculate, and they also do not interfere with sensation or orgasm [6]. The most feared complication is infection, although coated implants have lower infection rates [7]. A study of patient and partner satisfaction of two-part penile implants (such as the one our patient had) revealed good satisfaction for both parties [8]. We present the case of an elderly male with EO following a urinary injury from a penile prosthesis.

## Case presentation

A 79-year-old male presented to the emergency department due to left lower quadrant and left suprapubic area and left scrotum pain and dysuria that began four days prior to presentation. The patient had an inflatable penile prosthesis implant in place. The patient stated that a few months prior he went into urinary retention and had a traumatic catheter placed. The catheter placement was made more difficult due to his penile prosthesis. He ended up with a urethral injury. Since that time, he has had problems with frequent UTIs. He has benign prostatic hypertrophy for which he underwent transurethral resection of the prostate. He was being treated with cephalexin for a UTI. He denied any fevers, chills, chest pain, shortness of breath, nausea, vomiting, diarrhea, or headache. His past medical history was significant for hypertension, dyslipidemia, gastroesophageal reflux disease, squamous cell carcinoma, and melanoma. Past surgical history was significant for herniorrhaphy, penile implant, and knee surgery. He is an ex-smoker and non-drinker. His vital signs were as follows: blood pressure of 146/88 mmHg, temperature of 97.7°F, pulse of 77 beats per minute, and respiratory rate of 17 breaths per minute saturating at 97% on room air.

Physical examination was significant for abdominal and suprapubic tenderness and rebound. Genitourinary examination revealed scrotal swelling. Urinalysis revealed moderate blood and large leukoesterase. The remainder of the laboratory evaluation was unremarkable (Table 1).

**Table 1:** Lab results of patient and reference values

Tests	Results	Reference Ranges
Sodium (mmol/L)	138	135–145
Potassium (mmol/L)	4.4	3.5–5.3
Chloride	105	98–107
Carbon dioxide (mmol/L)	31	21–32
Anion gap (mmol/L)	2	4-12
Blood Urea Nitrogen (mg/dL)	15	7–18
Creatinine (mg/dL)	0.9	0.6–1.3
Estimated glomerular filtration rate	>60	>60
Glucose (mg/dL)	106	74–106
Calcium (mg/dL)	9.1	8.4–10.2
Total bilirubin (mg/dL)	0.7	0.0–1.0
Alanine transaminase (units/L)	31	15–37
Aspartate transaminase (units/L)	26	12–78
Total protein (g/dL)	7.4	6.4–8.2
Albumin (g/dL)	4.1	3.4–5.0
Lipase (Units/L)	71 L	73–393
Hematology		
White blood cells (x 10^3^/uL)	6.7	4.1–9.3
Red blood cells (x 10^6^/uL)	4.71	3.66–5.56
Hemoglobin (g/dL)	13.7 L	13.8–17.2
Hematocrit (%)	42.8	40.6–51.8%
Platelet count (x 10^3^/uL)	186	150–450
Absolute basophils (auto) (x 10^3^/uL)	0.1	0.00–0.20
Nucleated RBC (%)	0	0.0–0.0
Immature granulocytes (x 10^3^/uL)	0	0.00–0.00
Neutrophils (x 10^3^/uL)	3.9	1.4–6.5
Lymphocytes (x 10^3^/uL)	1.6	1.2–3.4
Monocytes (x 10^3^/uL)	0.9 H	0.1--0.6
Eosinophils (x 10^3^/uL)	0.3	0.00–0.70
Serology		
COVID-19	Negative	Negative
Urines		
Urine color	Yellow	Yellow
Urine appearance	Clear	Clear
Urine pH	6	5.0–8.0
Urine specific gravity	1.01	1.005–1.030
Urine protein (mg/dL)	Negative	Negative
Urine glucose (stick) (mg/dL)	Negative	Negative
Urine ketones (mg/dL)	Negative	Negative
Urine blood	Moderate H	Negative
Urine nitrate	Negative	Negative
Urine bilirubin	Negative	Negative
Urine urobilinogen (EU/dL)	0.2	0.2–1.0
Urine leukocyte esterase	Large H	Negative

**Figure 1:**
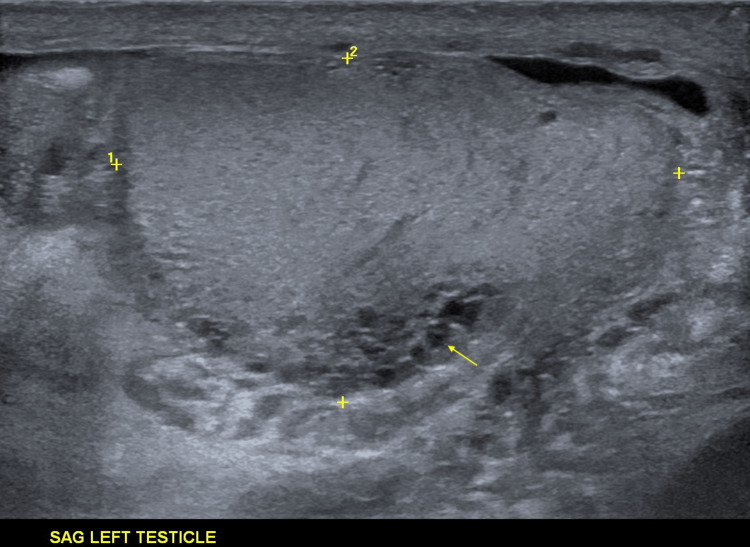
Varicocele in the left testicle (arrow).

Scrotal ultrasonography did not reveal any sonographic evidence of testicular torsion. Findings were compatible with left-sided EO. A moderate-sized complex left hydrocele was seen. Mild-to-moderate bilateral testicular atrophy was also noted. Heterogeneous testicular echogenicity may be on the basis of atrophy (Figure 1).

**Figure 2:**
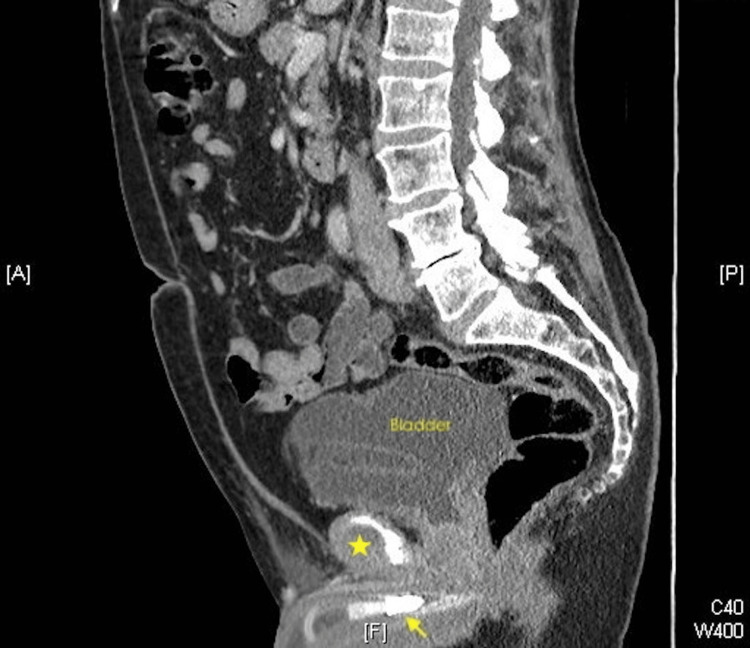
Computed tomography (sagittal view) scan demonstrating penile prosthesis (yellow arrow), and a loculated fluid collection (star).

Computed tomography scan of the abdomen and pelvis demonstrated a large well-loculated fluid collection in the right lower pelvic region adjacent to the bladder, surrounding the catheter of the penile prosthesis pump (Figure 2).

The patient was conservatively managed with intravenous antibiotics as per urology service’s advice and did not require any surgical intervention. He was discharged home the following day with outpatient follow-up.

## Discussion

Treatment of EO can be decided by the pathogens causing infection. For patients who are between 14 and 35 years of age, wherein gonococcal or chlamydial infection is probable, they should receive one dose of 250 mg ceftriaxone and 100 mg of doxycycline twice daily [9]. For patients whose infections can be attributed to coliform bacteria, mostly patients younger than 14 years and older than 35 years, 300 mg of ofloxacin twice per day for 10 days or 500 mg of levofloxacin once per day for 10 days is recommended [4].

In addition to antibiotics, it is recommended that patients limit their activity, use cold packs, and try scrotal elevation. Follow-up is suggested for up to seven days after the patient is evaluated and the initial treatment is given. Pain is expected to subside within one to three days, and patients who are older than 50 years should be evaluated for urethral obstruction secondary to prostatic hypertrophy [1]. the importance of completing the entire antibiotic treatment course should be emphasized to patients.

In a general population of patients who have EO, trauma resulting from urinary instrumentation is uncommon. A study involving 58 cases in Nigeria found that only three could be attributed to trauma/instrumentation (5.2%). While this etiology described only a few patients, the study ultimately suggested that in older men, urinary tract surgery and instrumentation are expected risk factors for EO and UTIs [10].

When EO is noted in the presence of a penile prosthesis, the concern for infection of the prosthesis itself should be considered. The most significant risk factors in this scenario are diabetes mellitus and revision surgery [11], neither of which our patient had. Furthermore, he did not evidence any systemic signs of sepsis (normal vital signs and no leukocytosis). In our patient’s case, the urology service decided on conservative management with a course of intravenous antibiotics. Had prosthesis removal been elected, the options would include immediate reimplantation of a new device (termed “concomitant implant after explant”) or revision surgery at a later date. The challenge with delayed reimplantation is fibrosis of the canal and shortening of the penis but is considered safer for the patient [12].

## Conclusions

Causes of EO often vary in patients based on age. Additional risk factors such as comorbidities, urinary tract instrumentation, and penile prostheses should also be taken into consideration. The mainstay of genitourinary infections is antibiotics targeted toward the infection, followed by consideration of implant removal when applicable.
